# Revealing the role of crystal orientation of protective layers for stable zinc anode

**DOI:** 10.1038/s41467-020-17752-x

**Published:** 2020-08-07

**Authors:** Qi Zhang, Jingyi Luan, Xiaobing Huang, Qi Wang, Dan Sun, Yougen Tang, Xiaobo Ji, Haiyan Wang

**Affiliations:** 1 Hunan Provincial Key Laboratory of Chemical Power Sources, College of Chemistry and Chemical Engineering, Central South University, Changsha, 410083 P. R. China; 2 College of Chemistry and Chemical Engineering, Hunan University of Arts and Science, Changde, 415000 P. R. China

**Keywords:** Energy, Materials chemistry, Batteries, Batteries

## Abstract

Rechargeable aqueous zinc-ion batteries are a promising candidate for next-generation energy storage devices. However, their practical application is limited by the severe safety issue caused by uncontrollable dendrite growth on zinc anodes. Here we develop faceted titanium dioxide with relatively low zinc affinity, which can restrict dendrite formation and homogenize zinc deposition when served as the protective layer on zinc anodes. The as-prepared zinc anodes can be stripped and plated steadily for more than 460 h with low voltage hysteresis and flat voltage plateau in symmetric cells. This work reveals the key role of crystal orientation in zinc affinity and its internal mechanism is suitable for various crystal materials applied in the surface modification of other metal anodes such as lithium and sodium.

## Abstract (web summary)

Zinc affinity plays a key role in the zinc plating and stripping processes but its internal mechanism is still unclear. Here, the authors report a protective layer with controllable zinc affinity by adjusting the crystal orientation to suppress the dendrite growth on the zinc anode interface.

## Introduction

Achieving higher energy density is the main development tendency for the next-generation battery system^1^. Metal anodes, such as lithium (Li), sodium (Na), and zinc (Zn), with high theoretical capacity and low electrochemical potential are considered as the most promising materials to meet this requirement^2,3^. However, the electrochemical performance of metal anodes is seriously affected by the dendrite formation during repeated charging and discharging. Metal dendrites are easily detached from anode mainbody and the newly exposed metal would react with electrolyte, leading to low Coulombic efficiency^4,5^. More seriously, separators may be punctured by the continuous dendrite growth, which directly causes the short circuit and poor cycle life of batteries^6,7^. Therefore, it is important to solve this issue for the industrialization of metal anodes.

Many approaches have been developed to achieve safer metal anodes, which mainly focus on two aspects: (1) improving metal deposition on current collectors. Nucleation can be facilitated by a three-dimensional structure with a uniform local electric field^8,9^. Some metal-affinity modification layers induced on current collectors are conducive to the adsorption of metal ions, which can guide the deposition and further alleviate dendrites^10–12^. (2) Optimizing the interface between metal and electrolyte. An artificial solid electrolyte interface (SEI) or an additional layer with inferior metal affinity can be applied as a protective layer to restrict dendrite growth^13,14^. Metal affinity is a key criterion to judge the applicable functions (guiding or restricting). However, taking titanium dioxide (TiO_2_) for example, it can be used both for modification of current collectors and protection of metal anodes according to previous reports^15–17^. These results seem contradictory because good metal affinity is required when TiO_2_ is used as a decoration on current collectors to homogenize metal deposition while low metal affinity is necessary if it is served as a protective layer^18,19^. TiO_2_ can be simultaneously applied to two different metal modification strategies with opposite requirements, indicating that there is an ambiguous internal mechanism affecting its metal affinity. Considering that exposed facets of a crystal have a great influence on catalytic activity, metal affinity may be also controllable by adjusting surface exposure^20,21^.

In this work, the interactions between Zn and different facets of TiO_2_ are first investigated by density functional theory (DFT) calculation and it is concluded that the (0 0 1) and (1 0 1) facets of TiO_2_ show relatively low Zn affinity. Accordingly, TiO_2_ with highly exposed (0 0 1) facet is prepared and applied as the protective layer for Zn metal anodes. The (0 0 1) faceted TiO_2_ layer can effectively prevent Zn dendrites from growing vertically and stabilize the interface between anode and electrolyte. As a result, the modified Zn anode exhibits long-term cycle life during Zn stripping and plating.

## Results

### Theoretical analysis and characterization of faceted TiO_2_

**Fig. 1:**
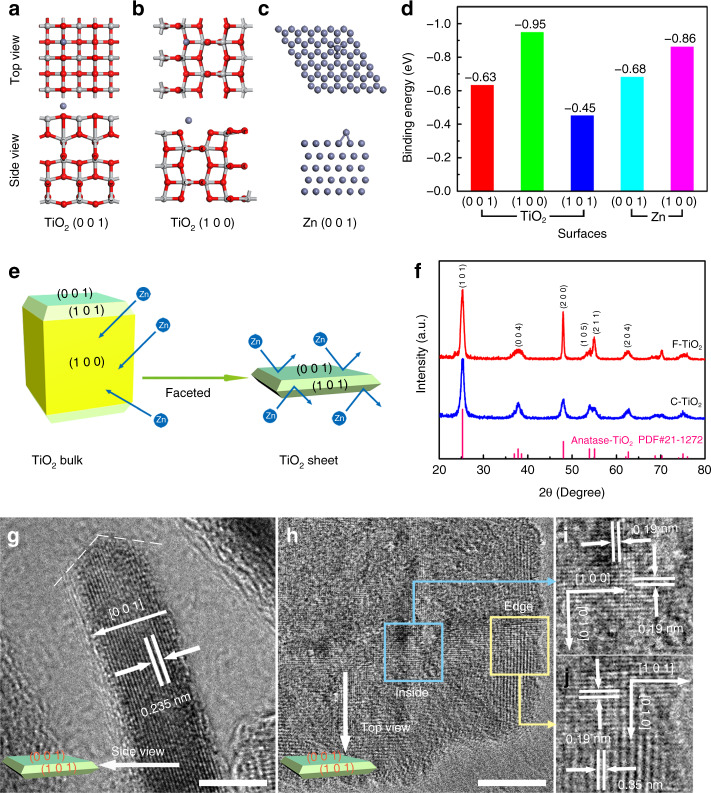
Theoretical simulation and characterization of F-TiO_2_. Calculations models of Zn absorbed on **a** TiO_2_ (0 0 1) facet, **b** TiO_2_ (1 0 0) facet, and **c** Zn (0 0 1) facet. **d** Calculated binding energies of Zn atom with different facets. **e** Schematic illustration of the interaction between Zn and anatase TiO_2_ with different exposed facets. **f** XRD patterns of F-TiO_2_ and C-TiO_2_. **g**–**j** HRTEM images of F-TiO_2_. Scale bars: 5 nm.

The mechanism for the interaction between Zn and different facets of TiO_2_ is first investigated by DFT calculation. As shown in Fig. 1a–c and Supplementary Figs. 1 and 2, the models of Zn atoms attached to TiO_2_ surfaces and Zn surfaces were constructed. The Zn affinity of TiO_2_ surfaces can be judged by comparing the binding energy of Zn atom attached to the TiO_2_ surface and Zn surface. It can be considered that a TiO_2_ facet is with high Zn affinity if the binding energy of Zn atom attached to the corresponding TiO_2_ facet is higher than that on the Zn surface. As summarized in Fig. 1d, the binding energy between Zn atom and TiO_2_ (1 0 0) facet is −0.95 eV, higher than that between Zn atom and Zn surfaces (−0.68 and −0.86 eV), indicating that Zn prefers to deposit on TiO_2_ (1 0 0) facet in comparison to Zn surface. It is detrimental for a protective layer since this priority can lead to the growth of Zn dendrites upon the layer and deactivate the protective effect. In contrast, there is a weaker absorption of Zn on TiO_2_ (0 0 1) and (1 0 1) facets with the binding energy of −0.63 and −0.45 eV, respectively, which is mainly because the more exposure of the lower coordinated Ti on these facets exhibits more intense repulsion to Zn atom^22^. The interaction between Zn and different TiO_2_ facets is illustrated in Fig. 1e. According to the above analysis, the facet orientation plays a key role in Zn affinity and suitable materials for a protective layer can be achieved by controlling the exposure of specific facet.

**Fig. 2:**
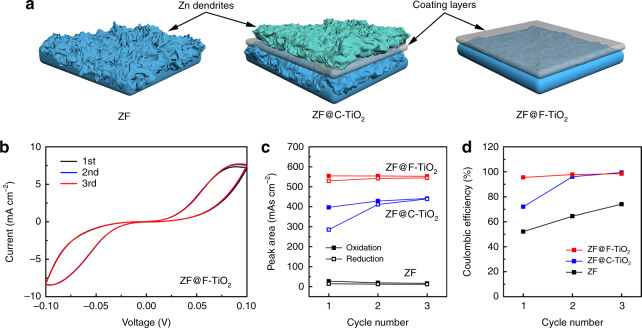
Zn deposition behavior of the prepared anodes. **a** Schematic illustration of the Zn plating process with different coating layers. **b** CV curves of Zn–Zn symmetric cells using ZF@F-TiO_2_ anode measured at 1 mV s^−1^. Peak areas of Zn stripping/plating reactions (**c**) and corresponding Coulombic efficiency (**d**) of the prepared Zn anodes in Zn–Zn symmetric cells.

As shown in Fig. 1f, the X-ray diffraction (XRD) patterns of the as-prepared faceted TiO_2_ (F-TiO_2_) and commercial TiO_2_ (C-TiO_2_) can be indexed to anatase TiO_2_. The average thickness along the growth direction of a crystal (*D*) can be calculated by Scherrer equation^23,24^:

1 $$D = \frac{{K\lambda }}{{\beta {\mathrm{cos}}\theta }}$$

where *K*, *λ*, *β*, and *θ* represent Scherrer constant, the wavelength of X-ray, full width at half maximum (FWHM) of diffraction peak and Bragg angle, respectively. For a specific crystal orientation, the larger FWHM in the XRD pattern indicates the smaller *D* in this direction, in other words, the larger exposed area of the corresponding facet (Supplementary Fig. 3)^25^. Accordingly, the broader (0 0 4) peak in F-TiO_2_ demonstrates the larger exposure of (0 0 1) facet and the narrower (2 0 0) peak F-TiO_2_ corresponds to the larger crystal size parallel to (0 0 1) facet, which is also the evidence of the relatively higher exposed area of (0 0 1) facet in comparison with C-TiO_2_^26^. In Raman spectra (Supplementary Fig. 4), the characteristic peaks of anatase TiO_2_ appear at 392.3 cm^−1^ (symmetric bending vibration, B_1g_), 513.7 cm^−1^ (antisymmetric bending vibration, A_1g_), 636.5 cm^−1^ (symmetric stretching vibration, E_g_)^27^. The 3-coordinated titanium (Ti) atoms on the (0 0 1) surface (Supplementary Fig. 5a) with lower coordination number than the 5-coordinated Ti atoms on (1 0 0) surface (Supplementary Fig. 5b) tend to show stronger bending vibration^28^. Therefore, the weaker A_1g_ and B_1g_ peaks in the Raman spectrum of F-TiO_2_ suggest its higher exposure of (0 0 1) facet. Transmission electron microscopy (TEM) images (Supplementary Fig. 6) clearly show the nanosheet structure of F-TiO_2_ with average width and thickness of 50 and 5 nm, respectively. From the side view of F-TiO_2_ nanosheet (Fig. 1g), lattice fringes with a lattice spacing of 0.235 nm are observed, which demonstrates that the [0 0 1] direction is perpendicular to the top surface. Figure 2h is the top-view high-resolution transmission electron microscopy (HRTEM) image of F-TiO_2_ nanosheet. There are orthogonal lattice fringes with equal lattice spacing (0.19 nm) inside the nanosheet (Fig. 1i), corresponding to [1 0 0] and [0 1 0] directions (both belong to <1 0 0> family of directions). Therefore, the normal direction of top surface is [0 0 1] direction perpendicular to both [1 0 0] and [0 1 0] directions^29^. According to the observation of side-view and top-view HRTEM images, it is confirmed that (0 0 1) facet is the highly exposed top surface of F-TiO_2_ nanosheet. Another set of orthogonal lattice fringes corresponding to [1 0 1] and [0 1 0] directions (Fig. 1j) indicates the existence of (1 0 −1) or (−1 0 1) facets (equivalent to (1 0 1) facet because of the tetragonal symmetry) at the edge of nanosheet^30^. Besides, the side surface intersects the top (0 0 1) surface at an obtuse angle (Fig. 1g and Supplementary Fig. 6c). It can be concluded that the side surface of F-TiO_2_ nanosheet is (1 0 1) facet rather than the vertical (1 0 0) facet. The percentage of the exposed (0 0 1) and (1 0 1) facets can be calculated to be 83% and 17%, respectively, by considering the nanosheet as a compressed square frustum. With regard to C-TiO_2_ (Supplementary Fig. 7), it exhibits a nanoparticle morphology with an average diameter of 20 nm and the irregular lattice fringes indicate its random growth orientation, which leads to more exposure of TiO_2_ (1 0 0) facets. The large area of extra TiO_2_ (1 0 0) facet is detrimental to prevent the growth of Zn dendrites. Accordingly, it is believed that F-TiO_2_ nanosheets with exposed (0 0 1) and (1 0 1) facets can completely shield Zn and restrict the formation of dendrites.

### Electrochemical performance of F-TiO_2_ as a protective layer

TiO_2_ protective layer was introduced on Zn anode by a simple blade coating method and the corresponding XRD pattern (Supplementary Fig. 8) demonstrates that the composite Zn anode is successfully synthesized. The Zn plating process on different Zn anodes is illustrated in Fig. 2a. Charges and ions tend to accumulate on the small tips at the surface of commercial Zn foil anodes when there is an impressed voltage. The resulting uneven interfacial electric field and ion concentration can induce preferential Zn growth and eventually lead to the formation of Zn dendrites during the repeated stripping and plating cycles^31^. When using C-TiO_2_ as the intermediate layer, Zn tends to grow on the surface of TiO_2_ layer with a higher Zn affinity. For comparison, the Zn plating reaction can be well confined under the protective layer and the smooth Zn layer can be deposited on the Zn anode by faceting the TiO_2_ to specific orientations with low Zn affinity. The cyclic voltammetry (CV) curves of F-TiO_2_ coated Zn foil (ZF@F-TiO_2_) in Zn–Zn symmetric cells (Fig. 2b) can almost maintain the identical shapes in comparison with the changed peaks of C-TiO_2_ coated Zn foil (ZF@C-TiO_2_) and Zn foil (ZF) (Supplementary Fig. 9), which indicates the superior reversibility of Zn stripping/plating in ZF@F-TiO_2_ anode. The peak areas of each redox reaction of CV curves can be obtained by integration operation (Fig. 2c). The larger peak area of ZF@F-TiO_2_ anode reflects the enhanced interfacial activity for Zn deposition^32^. Zn^2+^ driven by the electric field and concentration gradient migrate toward the Zn anode and tend to be repulsed by the F-TiO_2_ layer at the interface, leading to the Zn^2+^ accumulation near the anode surface. The increased Zn^2+^ concentration can not only activate more binding sites for Zn deposition but also accelerate the Zn^2+^ transfer rate on the surface to alleviate the uneven Zn^2+^ distribution, which is beneficial to the ordered interfacial Zn deposition^33,34^. Moreover, the highest Coulombic efficiency (reduction/oxidation) of ZF@F-TiO_2_ among all the prepared anodes (Fig. 2d) is also the evidence of its superior reversibility, which can be ascribed to the more uniform Zn deposition and less formation of “dead Zn”. TiO_2_ coated Zn foils exhibit better hydrophilicity than pure Zn foil (Supplementary Fig. 10), indicating that electrolyte can penetrate TiO_2_ layers to facilitate Zn^2+^ transport towards anode surface. The voltage profiles of the first ten cycles of zinc-stainless steel (Zn-SS) cells were recorded, which are shown in Supplementary Fig. 11. Pure Zn foil fails rapidly within 600 min due to the short circuit caused by the formation of dendrites. And the cycling stability of the cells is significantly improved when Zn foils are coated with the TiO_2_ interface layer. Specifically, ZF@F-TiO_2_ exhibits longer cycling life than ZF@C-TiO_2_, indicating its better Zn reversibility achieved by the specific exposed facets.

**Fig. 3:**
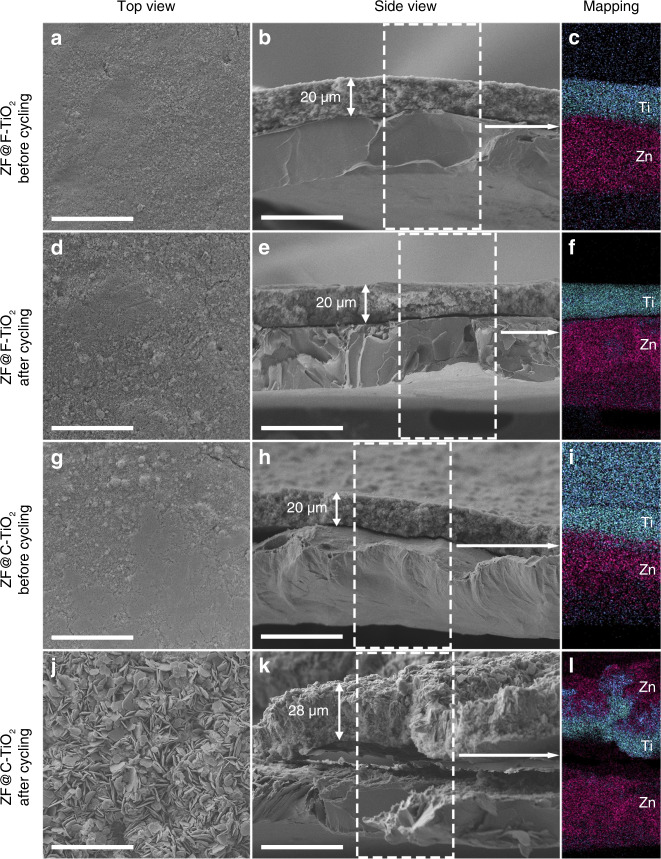
Morphology evolution of the prepared Zn anodes. SEM and the corresponding EDX mapping images of **a**–**f** ZF@F-TiO_2_ and **g**–**l** ZF@C-TiO_2_ before and after 10 cycles in Zn-SS cells. Scale bar: 40 μm.

Zn anodes were extracted from Zn-SS cells and their digital photographs are shown in Supplementary Fig. 12. Some unevenly distributed crystals are formed on the surface of ZF and ZF@C-TiO_2_, while ZF@F-TiO_2_ can maintain the original structure of F-TiO_2_ layer. Scanning electron microscope (SEM) images of these cycled electrodes were also compared with the fresh anodes (Fig. 3). The morphologies of ZF@F-TiO_2_ and ZF@C-TiO_2_ are consistent before cycling. TiO_2_ coating layer with a smooth surface and uniform thickness (20 μm) is in close contact with Zn foil. After cycling, there is no obvious change in ZF@F-TiO_2_ and its interface is still tightly combined. The well-defined distribution of Ti and Zn in the energy dispersive X-ray (EDX) mapping images (Fig. 3c, f) also demonstrates the good reversibility during stripping and plating cycles. As shown in Fig. 3j, k, Zn sheets (3μm in length) are observed on the surface of cycled ZF@C-TiO_2_, which is easily turned into Zn dendrites and causes the safety problem. C-TiO_2_ layer seems ineffective due to a large amount of Zn transferred from Zn foil and the formation of void space at the interface. The process of Zn transfer to the surface can be seen in the EDX mapping image (Fig. 3l). Supplementary Fig. 13 exhibits the more disordered surface with the wild growth of Zn dendrites on the cycled pure Zn foil, which is in agreement with the short-circuited Zn-SS cell within 10 cycles. From the different morphologies mentioned above, it is proved that the facet orientation plays an important role in adjusting Zn deposition behavior and TiO_2_ protective layer with highly exposed (0 0 1) facet with low Zn affinity can completely confine the Zn deposition in the restricted space. Besides, the TiO_2_ layer on the cycled anodes was removed by using methyl-2-pyrrolidinone (NMP) to dissolve polyvinylidene difluoride (PVDF) in the layers. As seen from the SEM images of the Zn surface after cycling (Supplementary Fig. 14), Zn deposition in ZF@F-TiO_2_ is flat and tends to accumulate parallel to the Zn surface, which also suggests the limited Zn growth by F-TiO_2_ protective layer with decreased Zn affinity.

**Fig. 4:**
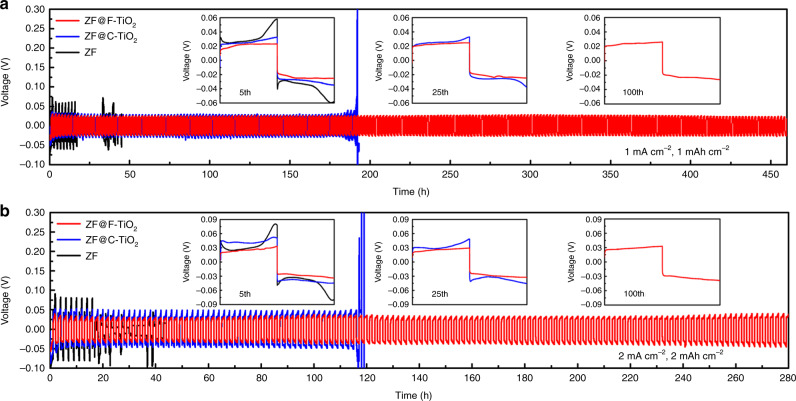
Electrochemical performance of prepared Zn anodes. Cycling performance Zn–Zn symmetric cells at **a** 1 mA cm^−2^ for 1 mAh cm^−2^ and **b** 2 mA cm^−2^ for 2 mAh cm^−2^.

The positive effect of the F-TiO_2_ layer on Zn plating behavior was further investigated by testing the cycling stability of Zn anodes in Zn/Zn symmetric cells. As shown in Fig. 4a, ZF@F-TiO_2_ can be operated steadily for more than 460 h at 1 mA cm^−2^ for 1 mAh cm^−2^, which is much superior to ZF@C-TiO_2_ (190 h) and ZF (20 h). When increasing the current density to 2 mA cm^−2^ and the specific capacity to 2 mAh cm^−2^ (Fig. 4b), ZF@F-TiO_2_ can still charge and discharge for 280 h in contrast to the quick failure of ZF@C-TiO_2_ and ZF with the shorter lifespan of 115 and 15 h, respectively. Besides, ZF@F-TiO_2_ exhibits the most stable voltage plateau and the lowest voltage hysteresis, reflecting the enhanced Zn transfer kinetics^35^. The electrochemical performance of ZF@F-TiO_2_ is competitive in comparison with several Zn anodes using protective coating materials (Supplementary Table 1). The full cells were assembled with the as-prepared Zn anodes and commercial manganese dioxide (MnO_2_) cathode. The full cell using ZF@C-TiO_2_ anode exhibits the lowest polarization voltage and best cycling performance with the capacity retention ratio of 84.1% after 300 cycles (Supplementary Fig. 15). The enhanced full cell performance using the F-TiO_2_ layer suggests its potential for practical application.

## Discussion

High-performance Zn anode without external dendrite growth was fabricated by coating TiO_2_ with highly exposed (0 0 1) facet on commercial Zn foil. Benefiting from the specific crystal orientation of TiO_2_ with abomination to Zn absorption, Zn^2+^ transferred by the electric field was enriched on the anode surface. Thus, the increased interfacial Zn^2+^ concentration could induce uniform nucleation and the further Zn deposition was guided to grow laterally. The as-prepared Zn anode exhibited superior Zn stripping and plating performance with a long lifespan (460 h at 1 mA cm^−2^ for 1 mAh cm^−2^). More importantly, the strategy to change the Zn affinity by adjusting the exposure of the facet provides a deep insight into the internal mechanism of metal affinity and can be extended to interfacial modification for other metal anodes.

## Methods

### Synthesis of F-TiO_2_

Tetrabutyl titanate (10 mL, Aladdin) and hydrofluoric acid (1.2 mL, 40 wt%, Sinopharm) were added into a 50 mL Teflon-lined autoclave and then maintained at 180 °C for 24 h. The precipitates were collected by vacuum filtration then washed by ethanol and dried at 80 °C for 6 h.

### Fabrication of ZF@F-TiO_2_

F-TiO_2_ and PVDF were mixed in a weight ratio of 9:1 with NMP as the dispersant. The slurry was pasted onto Zn foil (30 μm) and dried at 80 °C for 24 h under vacuum. For comparison, ZF@C-TiO_2_ was also prepared by replacing F-TiO_2_ with C-TiO_2_.

### Characterizations

XRD was tested by a Bruker D8 X-ray diffractometer with monochromatized Cu Kα radiation (wavelength = 1.5406 Å). Raman spectra were recorded using a Renishaw inVia spectrometer using an excitation wavelength of 532 nm. Morphologies of the prepared TiO_2_ and Zn anodes were characterized by SEM (Nova NanoSEM 230) and TEM (Tecnai G2 F20 S-TWIN). The contact angle was measured by a drop shape analyzer (DSA100).

### Electrochemical measurements

Coin-type cells (CR2025) were assembled for Zn–Zn symmetric cells, Zn-SS half cells and Zn-MnO_2_ full cells with glass fiber as separator and 1 M zinc sulfate (ZnSO_4_) aqueous solution as the electrolyte. Battery performance was evaluated using a Neware battery testing system. CV measurement for Zn–Zn symmetric cells was conducted on a CHI760E electrochemical workstation in the voltage range of −0.1–0.1 V. Zn-SS half cells were cycled with a specific capacity of 1  mAh cm^−2^ at 1 mA cm^−2^ for the charging process and a cut-off potential of −0.3 V at 1 mA cm^−2^ for the discharging process. Full cells were cycled between 1.0 and 2.0 V using commercial MnO_2_ (Macklin) as the cathode. In all, 0.1 M manganese sulfate (MnSO_4_) was added in the ZnSO_4_ electrolyte to prevent Mn^2+^ dissolution.

### Computational details

The first-principles calculations were conducted using generalized gradient approximation (GGA) and Perdew–Burke–Ernzerhof (PBE) exchange-correlation functional in DMol3 module of Materials Studio (version 8.0) of Accelrys Inc. An all-electron numerical basis set with polarization functions (DNP basis set) and a DFT-D method within the Grimme scheme was employed. The convergence tolerance was set to 1.0 × 10^−5^ Ha (1 Ha = 27.21 eV) for energy, 2.0 × 10^−3^ Ha Å^−1^ for maximum force and 5.0 × 10^−3^ Å for maximum displacement. Common facets of TiO_2_ and Zn were investigated in this simulation, including TiO_2_ (0 0 1), TiO_2_ (1 0 0), TiO_2_ (1 0 1), Zn (0 0 1) and Zn (1 0 0). Each facet was set as a five-layer 3 × 3 supercell with top three-layer atoms releasable. Zn atom was placed in the vertex of the oxygen octahedron of the TiO_2_ facet or the tetrahedral vertex of the Zn facet before geometry optimization. Binding energy (*E*_b_) was calculated by the following equation:

2 $$E_{\mathrm{b}} = E_{{\mathrm{total}}} - E_{{\mathrm{sub}}}-E_{{\mathrm{Zn}}}$$

*E*_total_, *E*_sub_, and *E*_Zn_ represent the total energy of the facet combined with Zn atom, the energy of the facet and the energy of Zn atom, respectively.

## Supplementary information

Supplementary Information

## Data Availability

The data sets generated and/or analyzed in this study are available from the corresponding author on reasonable request.
