# Supplementary material for: Revealing the role of crystal orientation of protective layers for stable zinc anode
Source: Nat Commun. 2020 Aug 7;11:3961. doi: 10.1038/s41467-020-17752-x (PMC7415142; doi:10.1038/s41467-020-17752-x)
Supplement: Supplementary file 1 — Supplementary Information [file 41467_2020_17752_MOESM1_ESM.pdf]

Supplementary Information

Revealing the role of crystal orientation of protective layers for stable zinc anode

Zhang et al.

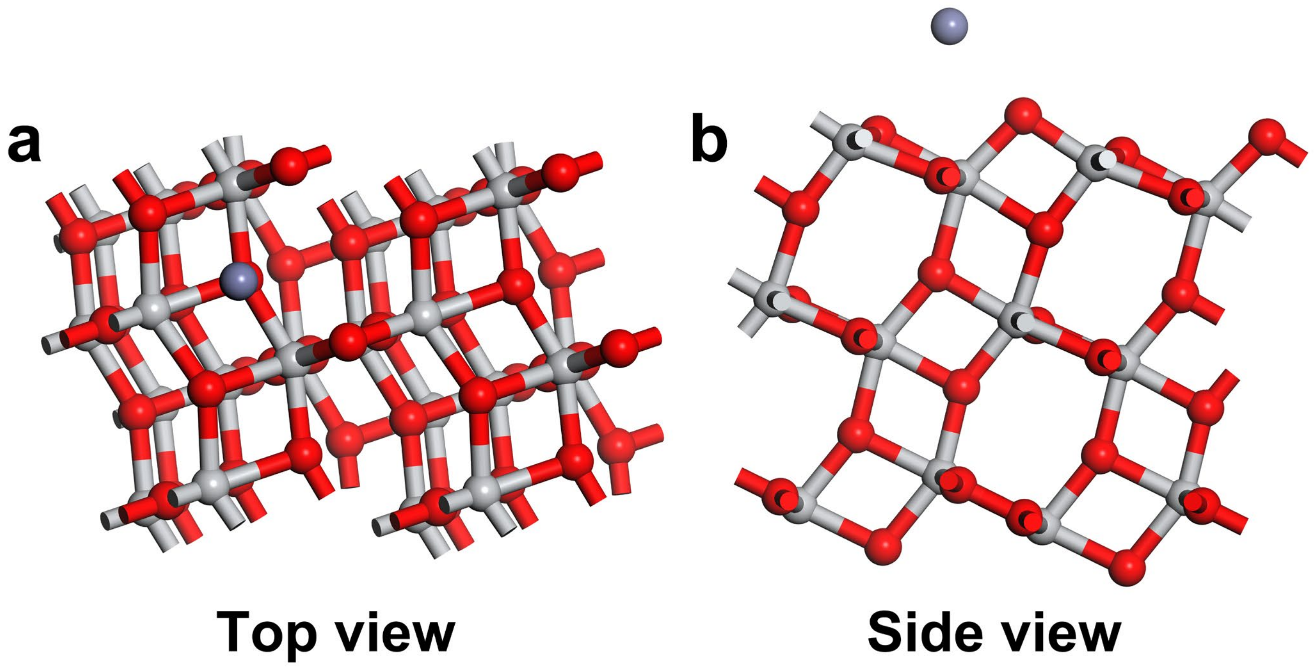

Supplementary Fig. 1 Top view **(a)** and side view **(b)** of the calculation model of Zn absorbed on the TiO_2 (1 0 1) facet.

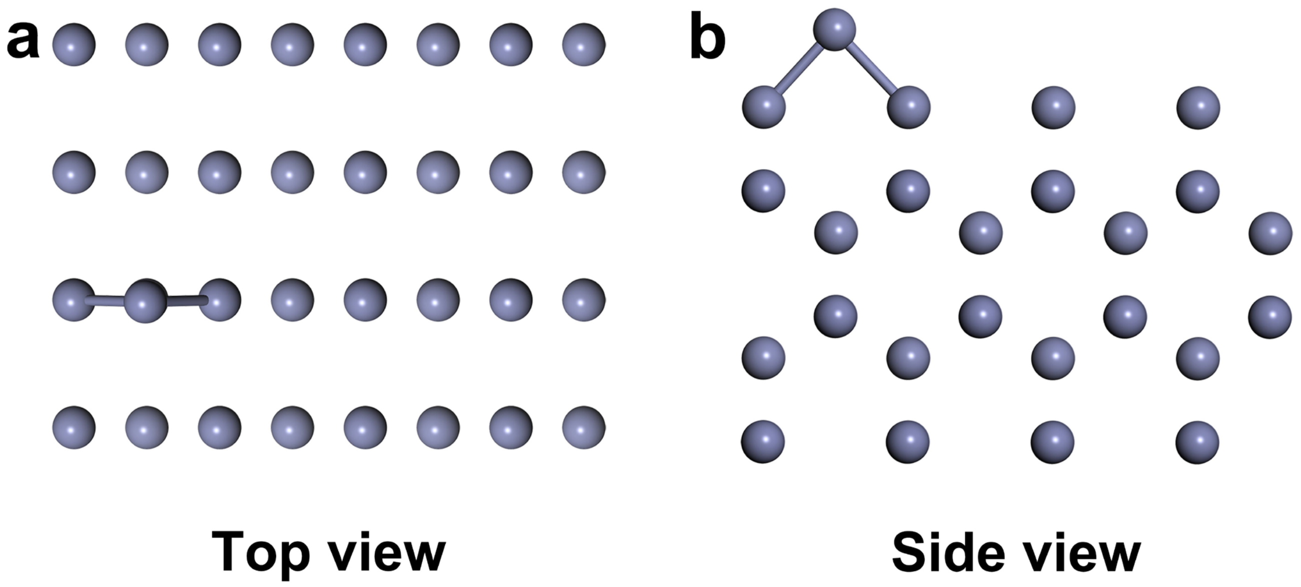

Supplementary Fig. 2 Top view **(a)** and side view **(b)** of the calculation model of Zn absorbed on the Zn (1 0 0) facet.

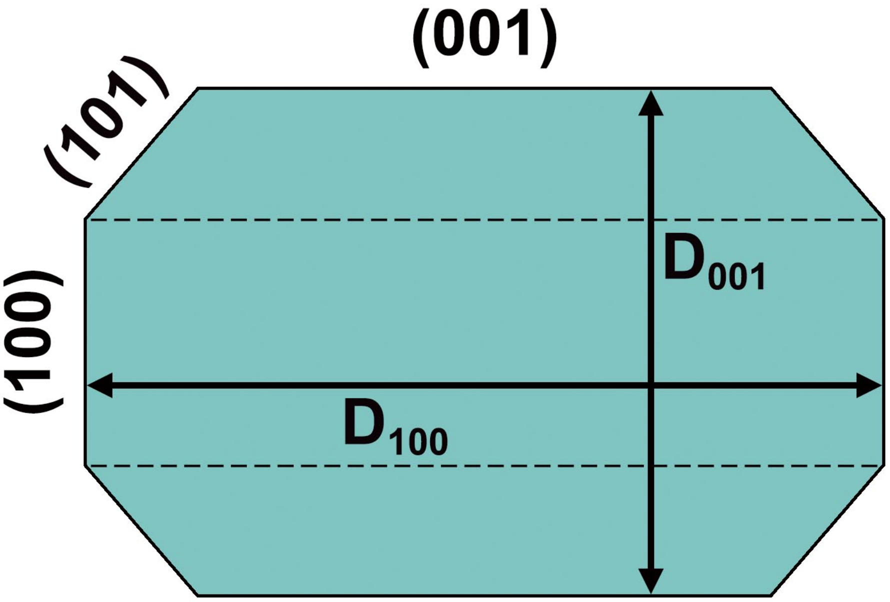

Supplementary Fig. 3 Crystal model to illustrate the relationship between crystal thickness and facet exposure.

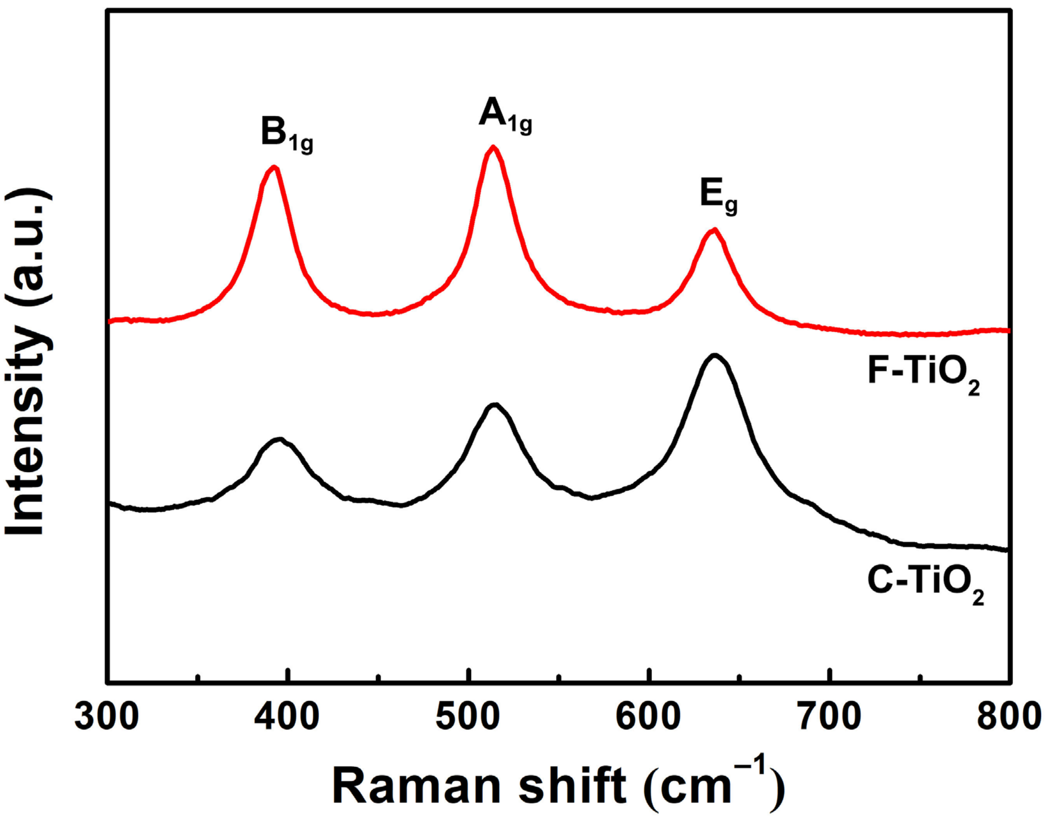

Supplementary Fig. 4 Raman spectra of F-TiO_2 and C-TiO_2.

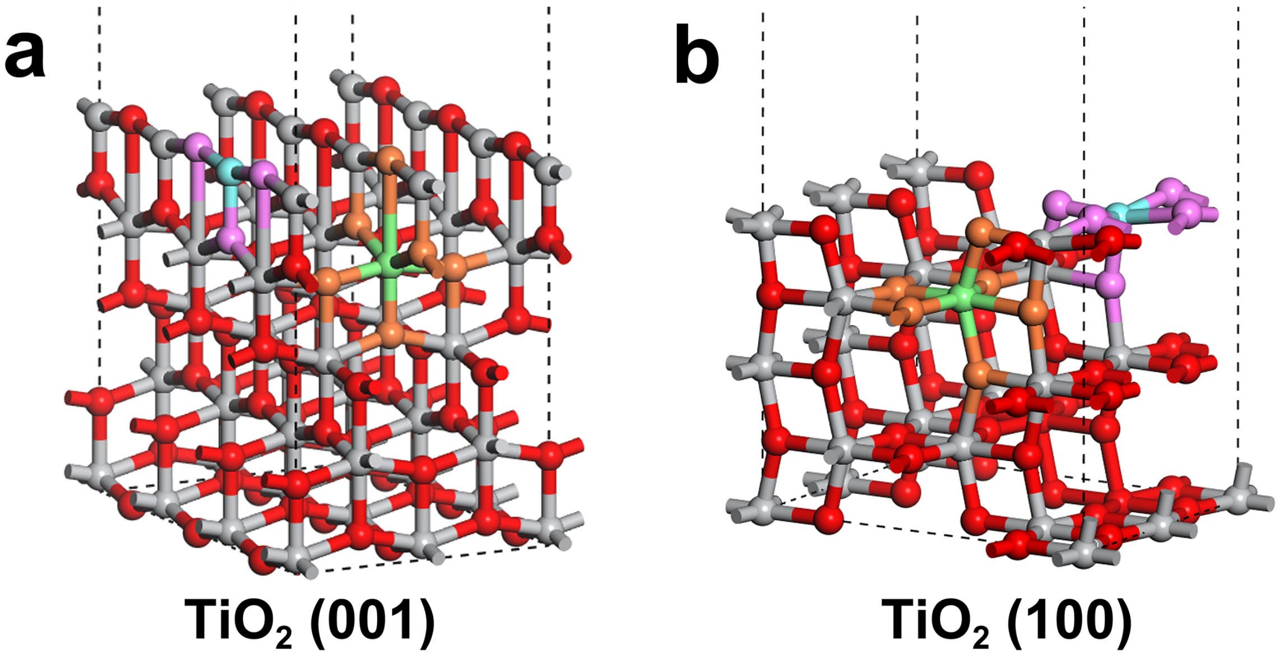

Supplementary Fig. 5 Structure models of **(a)** TiO_2 (0 0 1) facet and **(b)** TiO_2 (1 0 0) facet. Surface Ti atoms, inner Ti atoms, O atoms coordinated with surface Ti atoms and O atoms coordinated with inner Ti atoms are marked with blue, green, pink and orange, respectively.

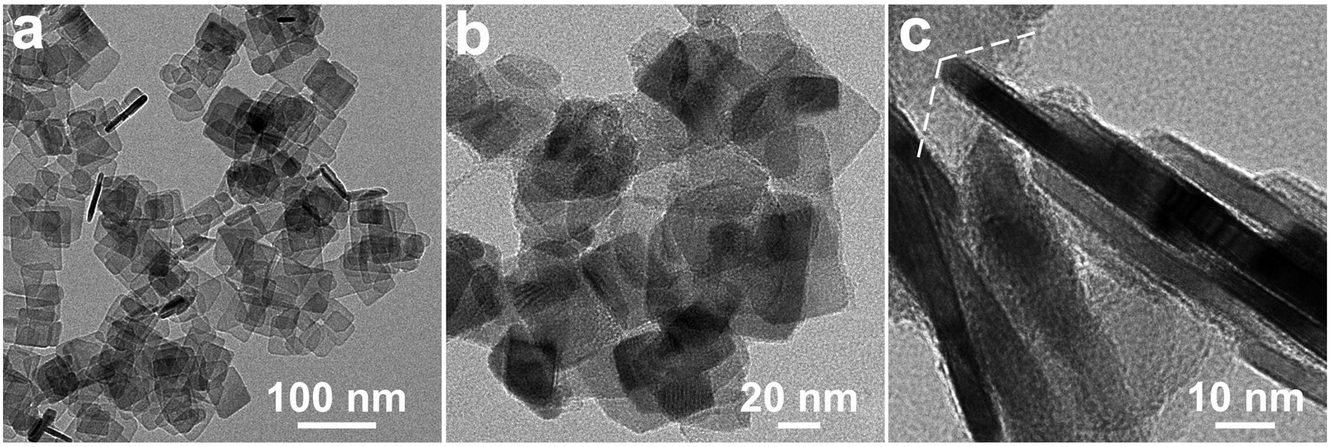

Supplementary Fig. 6 TEM images of F-TiO_2 from top view **(a, b)** and side view **(c)**.

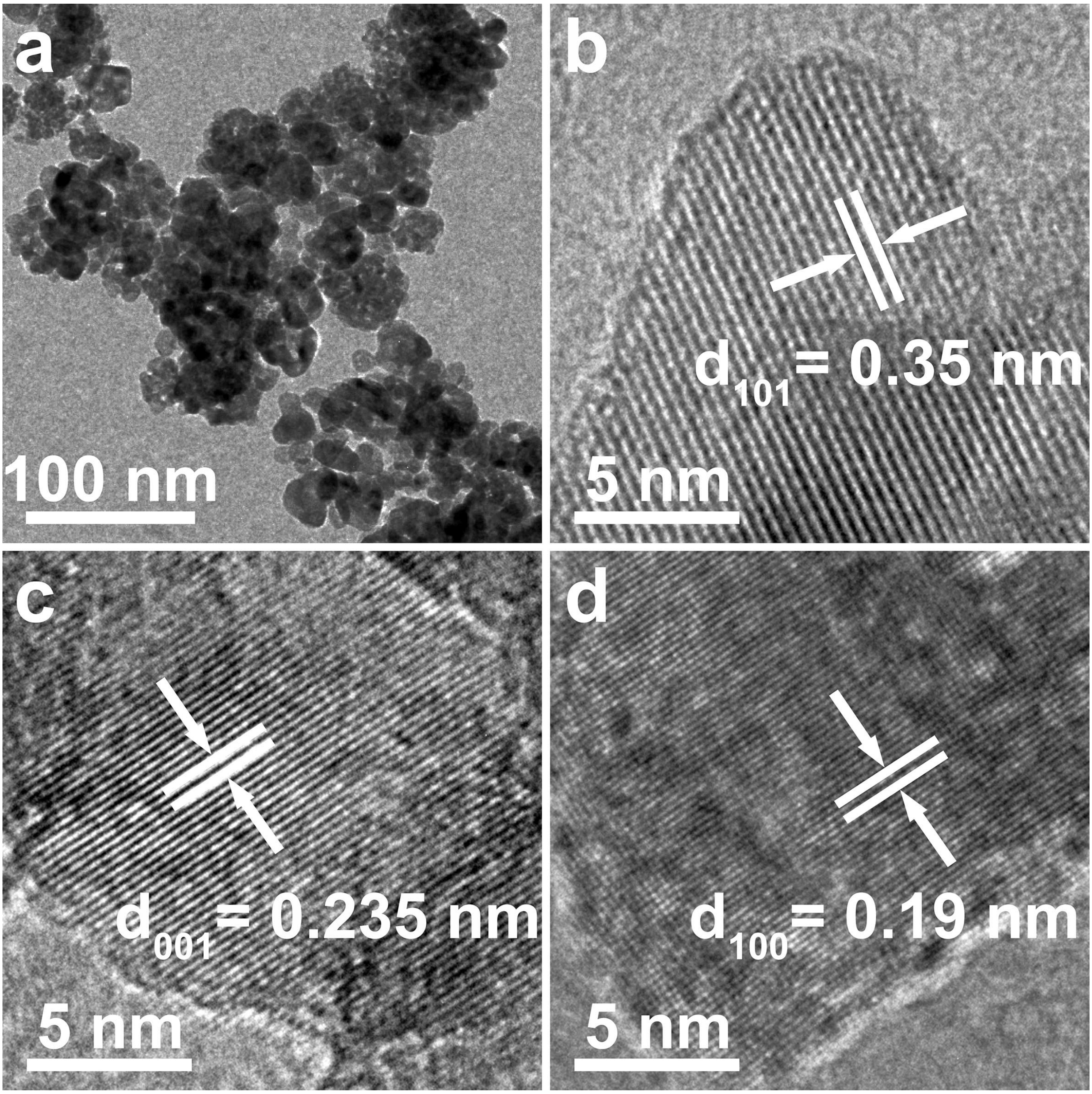

Supplementary Fig. 7 TEM **(a)** and HRTEM **(b-d)** images of C-TiO_2.

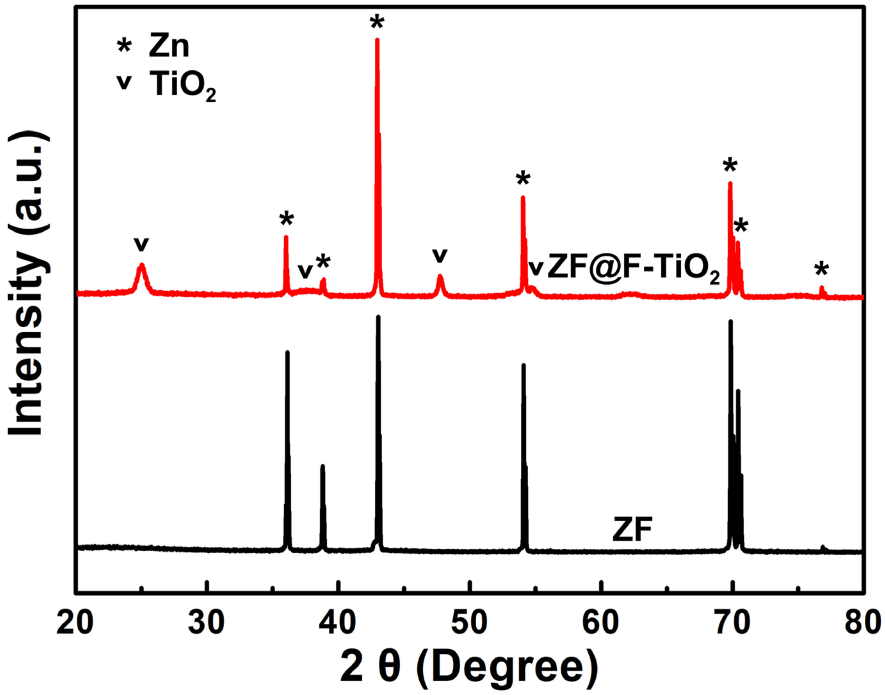

Supplementary Fig. 8 XRD patterns of ZF@F-TiO_2 and ZF.

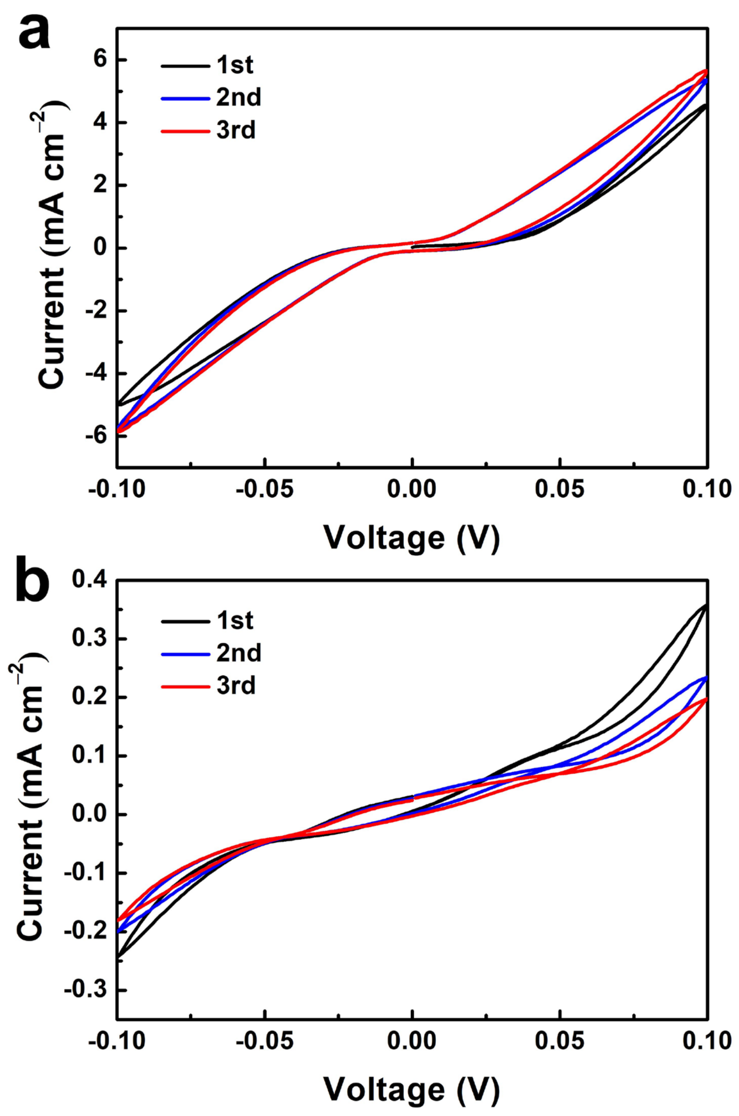

Supplementary Fig. 9 CV curves of Zn-Zn symmetric cells using **(a)** ZF@C-TiO_2 and **(b)** ZF.

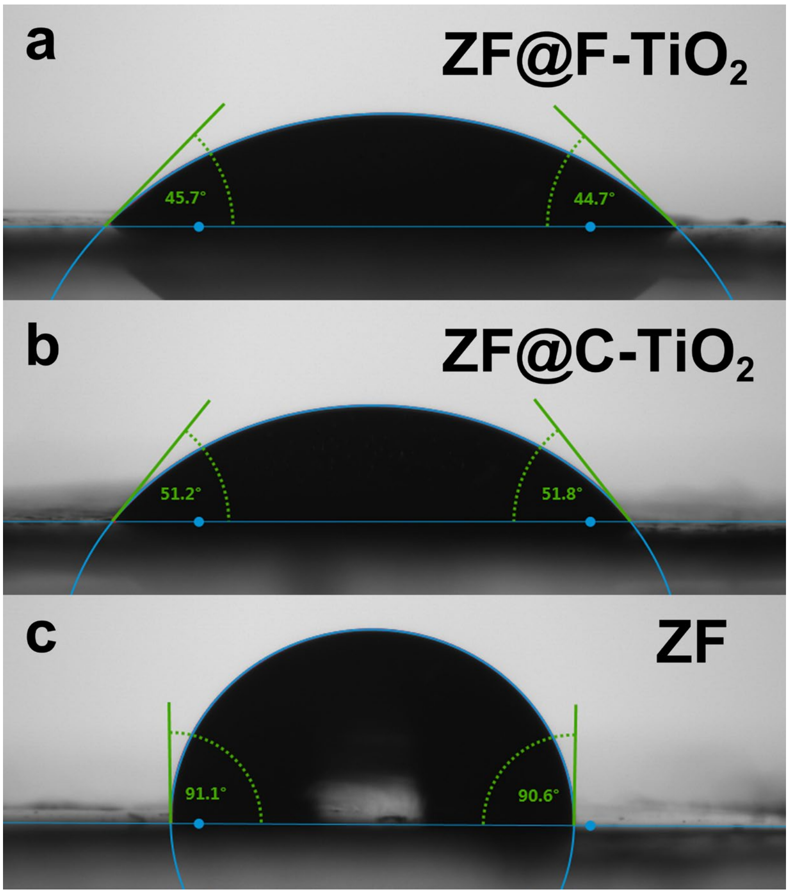

Supplementary Fig. 10 Contact angles of $ZnSO_4$ electrolyte on **(a)** ZF@F-TiO_2, **(b)** ZF@F-TiO_2 and **(c)** ZF.

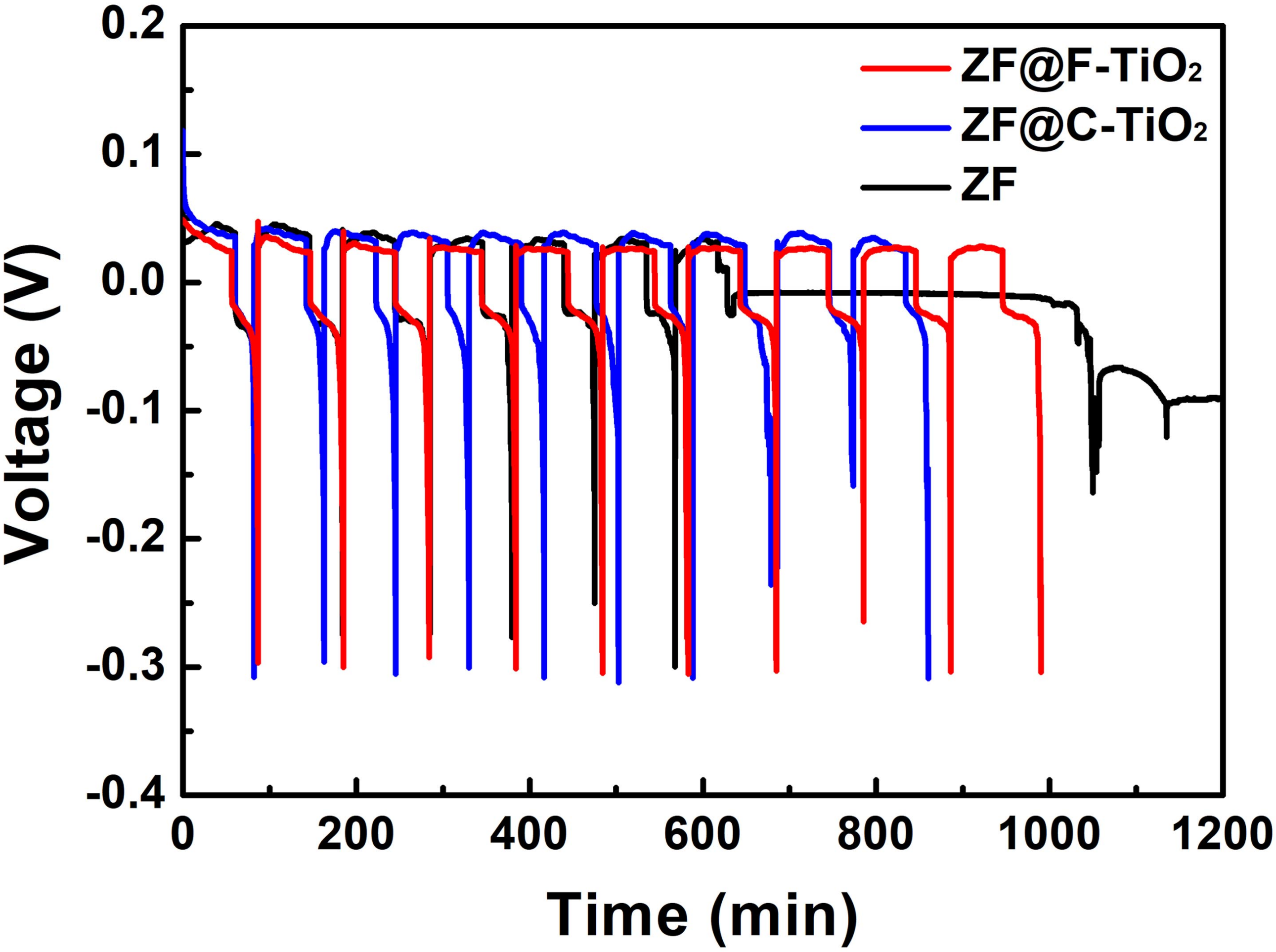

Supplementary Fig. 11 Voltage profiles of Zn-SS cells cycled at 1mA cm^{-2} for 1 mAh cm^{-2}.

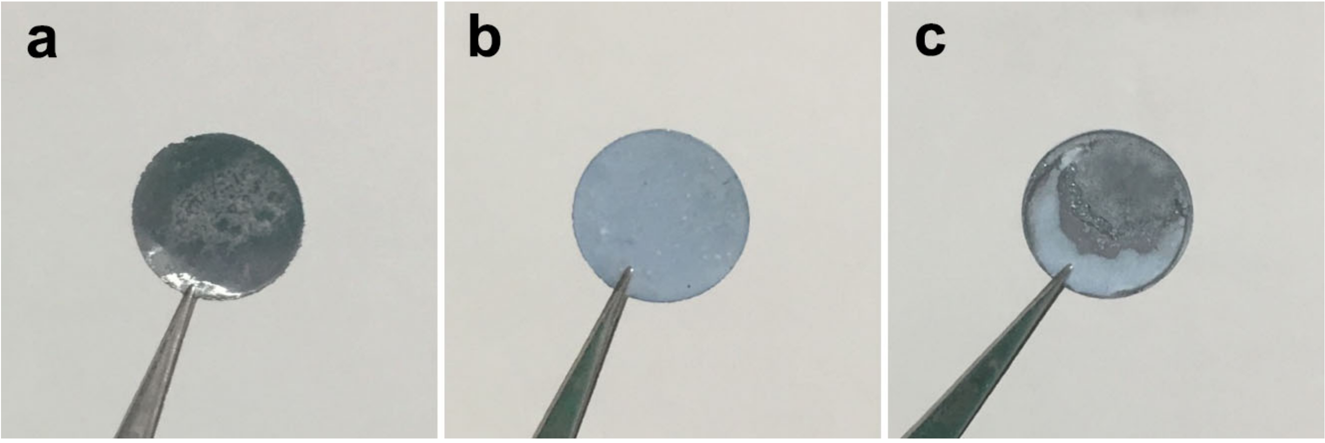

Supplementary Fig. 12 Digital photographs of **(a)** ZF, **(b)** ZF@F-TiO_2 and **(c)** ZF@C-TiO_2 after cycling.

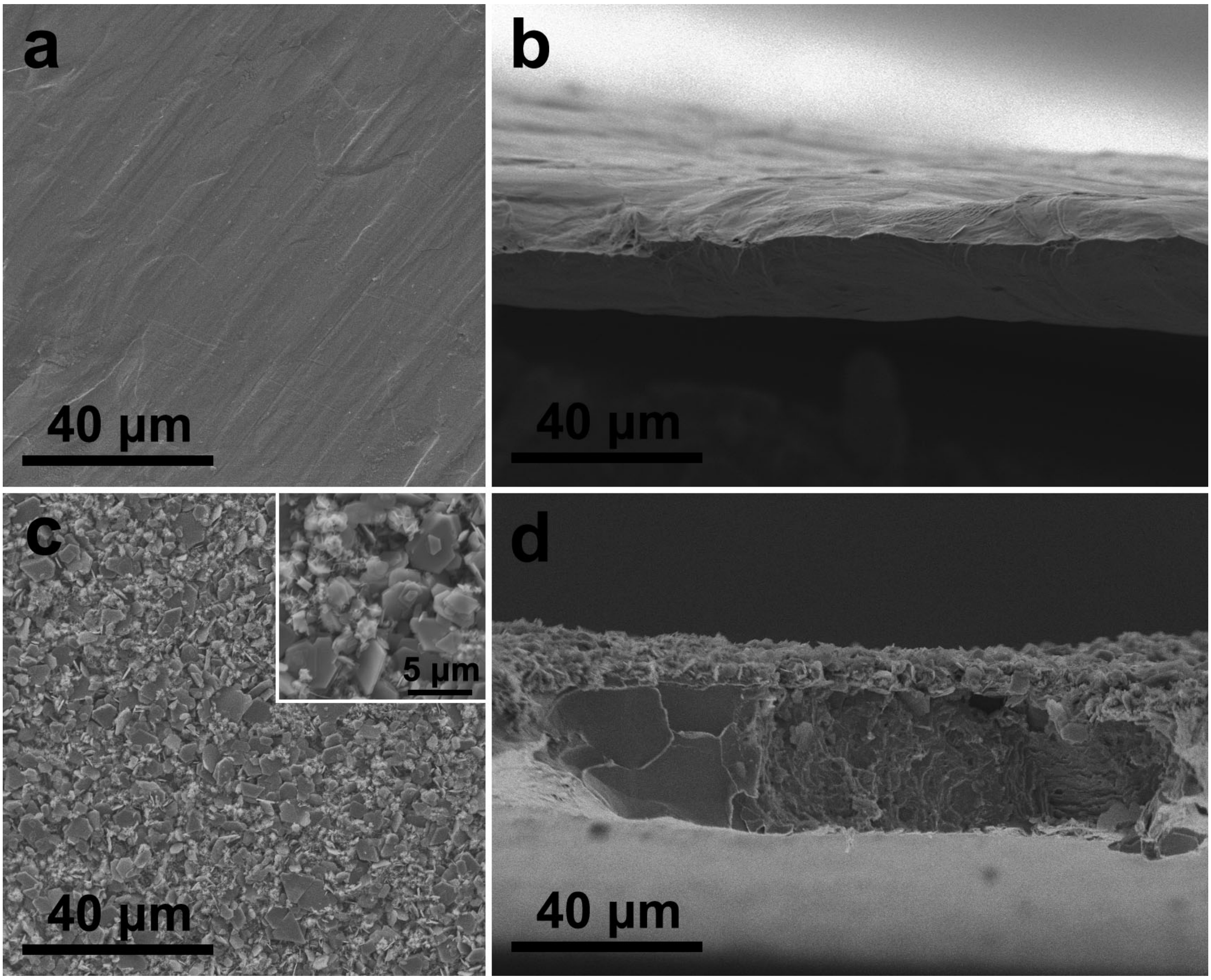

Supplementary Fig. 13 SEM images of ZF@F-TiO_2 **(a, b)** before and **(c, d)** after cycling.

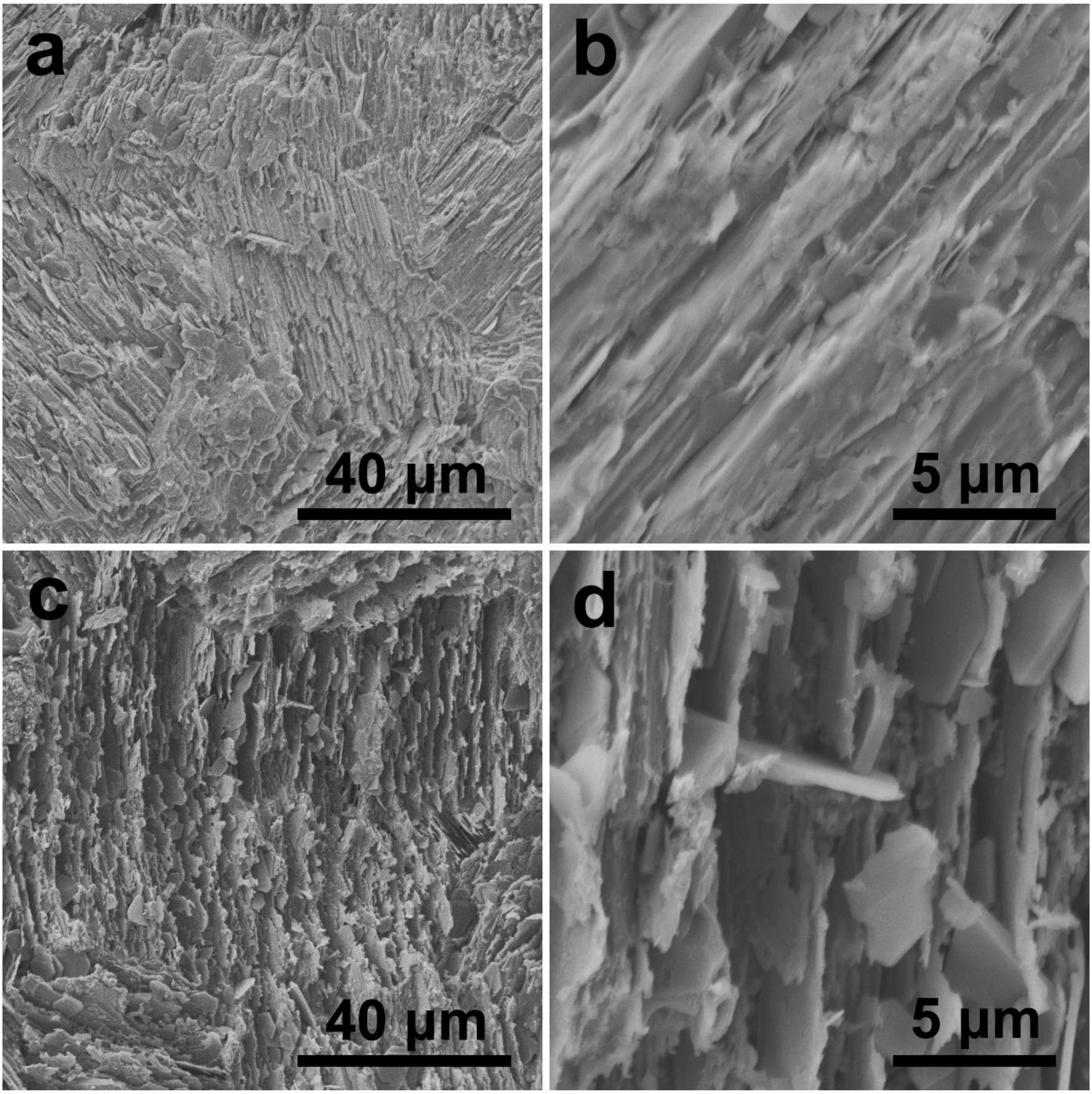

Supplementary Fig. 14 SEM images of Zn surface after cycling using **(a, b)** C-TiO_2 and **(c, d)** F-TiO_2 as the coating layer.

Supplementary Table 1 Comparison of recently reported aqueous Zn anode using protective coating materials.

Coating material	Electrolyte	Cycle life	Voltage hysteresis	Ref.
**Nanoporous $CaCO_3$**	3 M $ZnSO_4$	800 h at 0.25 mA cm^{-2} for 0.05 mAh cm^{-2}	80 mV	[1]
**Reduced graphene oxide**	1 M $ZnSO_4$	200 h at 1 mA cm^{-2} for 2 mAh cm^{-2}	52 mV	[2]
**Carbon nanotubes**	2 M $ZnSO_4$	400 h at 0.5 mA cm^{-2} for 0.15 mAh cm^{-2}	80 mV	[3]
**nanoporous ZnO**	2 M $ZnSO_4$	500 h at 5 mA cm^{-2} for 1.25 mAh cm^{-2}	43 mV	[4]
**Polyacrylonitrile membrane**	2 M $ZnSO_4$	350 h at 0.5 mA cm^{-2} for 0.25 mAh cm^{-2}	40 mV	[5]
**Faceted TiO_2**	1 M $ZnSO_4$	460 h at 1 mA cm^{-2} for 1 mAh cm^{-2}	42 mV	This work

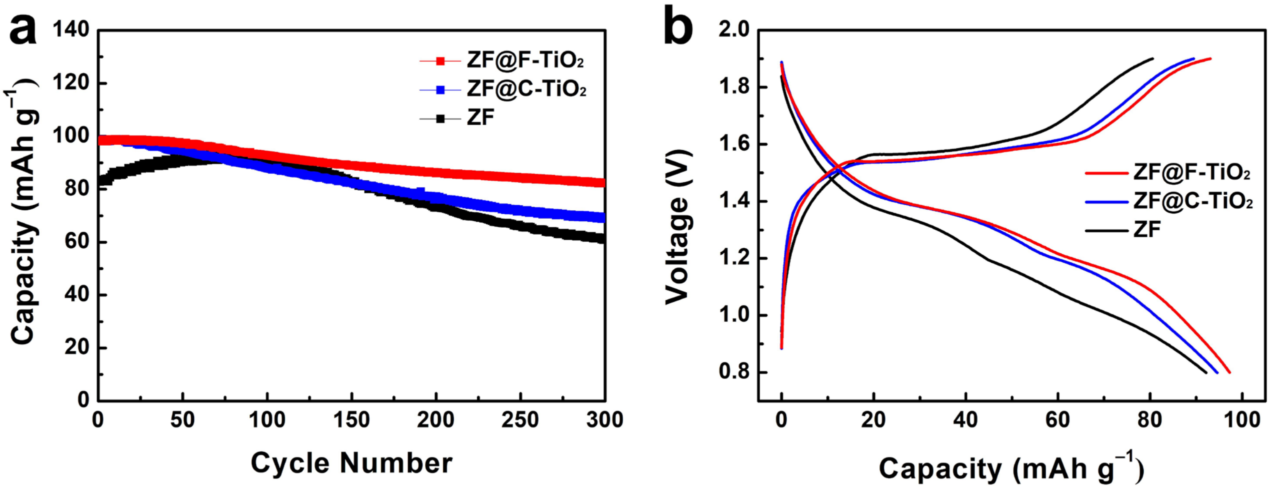

Supplementary Fig. 15 a Cycling performance comparison of the Zn-MnO_2 full cells at 1000 mA g^{-1}.

b The charge-discharge curves in the 50th cycle.

Supplementary References

1. Kang L, *et al*. Nanoporous $CaCO_3$ coatings enabled uniform Zn stripping/plating for long-life zinc rechargeable aqueous batteries. *Adv. Energy Mater.* **8**, 1801090 (2018).

2. Shen C, *et al.* Graphene-boosted, high-performance aqueous Zn-ion battery. *ACS Appl. Mater. Interfaces* **10**, 25446-25453 (2018).

3. Li M, *et al*. A novel dendrite-Free Mn^{2+}/Zn^{2+} hybrid battery with 2.3 v voltage window and 11000-cycle lifespan. *Adv. Energy Mater.* **9**, 1901469 (2019).

4. Xie X, *et al*. Manipulating the ion-transfer kinetics and interface stability for high-performance zinc metal anodes. *Energy Environ. Sci.* **13**, 503-510 (2020).

5. Lee B, *et al*. Dendrite suppression membranes for rechargeable zinc batteries. *ACS Appl. Mater. Interfaces* **10**, 38928-38935 (2018).
